# Efficacy comparison of different acupuncture methods for herpes zoster: A Bayesian analysis protocol

**DOI:** 10.1097/MD.0000000000020833

**Published:** 2020-06-26

**Authors:** Huachong Xu, Yucong Shi, Pei Liu, Li Deng

**Affiliations:** College of Traditional Chinese Medicine, Jinan University, Guangzhou, Guangdong, China.

**Keywords:** acupuncture, Bayesian analysis, herpes zoster, moxibustion

## Abstract

**Background:**

Acupuncture methods (include moxibustion) are used frequently in the treatment of herpes zoster. However, the choice is usually made only based on personal experience among different acupuncture methods. This study aims to compare the efficacy of different acupuncture methods for herpes zoster.

**Methods:**

All randomized controlled trials of acupuncture methods for herpes zoster will be searched in 7 databases including Cochrane Library, Embase, PubMed, Web of Science, Wan-fang database, China National Knowledge Infrastructure database, and VIP Chinese Science and Technique Journals database database. After screening process, effectiveness rate will be extracted from all the included randomized controlled trials as primary outcomes. The Bayesian network meta-analysis will be conducted by generate mixed treatment comparisons 0.14.3, Stata13.0, and Review Man 5.3.

**Results:**

The results of this study will be submitted to a peer-reviewed journal for publication.

**Conclusions:**

Our review will compare the efficacy of different acupuncture treatments for herpes zoster and find a better selection guideline for clinicians and patients.

**PROSPERO registration number:**

CRD42020175189.

## 1 Introduction

Herpes zoster is a neuro-cutaneous disease, usually resulting from the reactivation of varicella-zoster virus which remains dormant in the sensory ganglia following primary infection (chickenpox).^[1,2]^ Herpes zoster causes the characteristic vesicular skin rash localized in the sensory region of the affected ganglia, and is often accompanied by severe pain, pustular skin lesions, or itching.^[3]^ Approximately 25% of the world's population have the risk of developing herpes zoster during their lifetime, and the incidence of herpes zoster has been shown to be strongly related to age.^[4–6]^ Two-thirds of all shingles patients are over the age of 50 due to decreasing varicella zoster virus specific cell-mediated immunity with advancing age.^[7]^ As a common disease, herpes zoster has a great impact on the quality of life especially due to the pain during the acute phase and post-herpetic neuralgia.^[8–10]^ Most of the patients suffer a great psychological and economic burden.^[10]^

The aim of herpes zoster treatment is to relieve pain in the acute phase, to limit the spread and duration of skin lesions and to prevent or alleviate post-herpetic neuralgia and other acute and chronic complications. Treatments for herpes zoster include local therapy and systemic therapy. Systemic therapy is more commonly used with antiviral therapy, corticosteroid therapy, neuralgia therapy, and neurotrophic therapy because local therapy have limited effect.^[3]^ Currently, medications for antiviral therapy, including acyclovir, valaciclovir, and famciclovir, can alleviate the symptoms and reduce healing time,^[3,11]^ but often cause some adverse reactions. Additionally, it is hard to receive a satisfactory effect for the elderly with reduced renal function or renal insufficiency patients due to the limited dosage and cumulative toxicity.

As a safe and natural therapy, acupuncture and moxibustion treatments are considered as an effective alternative treatment and recommended in Chinese medicine clinical practice guidelines for treating the acute symptoms of herpes zoster in China.^[12]^ Some clinical studies^[13,14]^ and systematic analysis^[15–18]^ have shown that acupuncture treatments, alone or combine with drugs, may have a better therapeutic effect than simple drug therapy in pain reduction and skin conditions of herpes zoster. There are various acupuncture treatments used for herpes zoster in the clinic, including fire-acupuncture, surround-acupuncture, moxibustion, body acupuncture, electro-acupuncture, combination treatments, and so on. However, the previous studies^[15–18]^ only explored the effectiveness between 1 acupuncture treatment and a control intervention or only studied its effectiveness considering all the acupuncture treatments as a whole. As lacking of authoritative evidence and guidelines, the choice of different acupuncture therapies is usually made basing on the experience of clinical doctors, for which may lead to an unsatisfactory effect and longer course of treatment. Overall, the comparison of the efficacy among various acupuncture therapies is urgently need a detailed and in-depth exploration.

Compared to previous meta-analysis, recent researches have been incorporated into a network meta-analysis to perform a critical evaluation for the commonly used methods of acupuncture and moxibustion in herpes zoster. Our review aims to compare the effectiveness of different acupuncture treatments for herpes zoster and finding a better selection guideline for clinicians and patients.

## 2 Methods

Ethical approval is not necessary for this systematic review.

### 2.1 Search Strategy

To identify eligible randomized controlled trials for this network meta-analysis, we will search 7 electronic databases systematically from inception to December 31, 2019, including Cochrane Library, Embase, PubMed, Web of Science, Wan-fang database, China National Knowledge Infrastructure database, and VIP Chinese Science and Technique Journals database. The following search terms were conducted: (“Acupuncture” OR “Acupuncture Therapy” OR “Moxibustion”) AND (“Herpes Zoster” OR “Shingles” OR “Herpes zoster Virus” OR “Varicella Zoster Virus Infection”) AND (“Randomized Controlled Trial” OR “Controlled Clinical Trial” OR “Randomized” OR “Controlled”).

### 2.2 Inclusion criteria

#### 2.2.1 Types of trials

Clinical randomized controlled trials will be selected, which were published in the journal with English and Chinese.^[19]^

#### 2.2.2 Participants

Participants should have a primary diagnosis of herpes zoster according to diagnostic criteria or clinical diagnosis, regardless of age, gender, and disease duration. The main diagnostic criteria for herpes zoster include the following: Standards for Diagnosis and Curative Effect of Chinese Medical Symptom; Dermatovenerology; Modern dermatology; Cecil's medicine; Internal medicine; Chinese clinical dermatology; Diagnosis of conventional.

The detailed diagnostic standards are as follows^[20]^:

(1) skin lesions are mostly blisters the size of mung beans, which are clustered with relatively tense blisters with red basal base and usually unilateral distribution and arranged into ribbons. In severe cases, the lesion may be hemorrhagic or gangrenous;
(2) The rash is usually preceded by a tingling or burning sensation of the skin, which may be accompanied by mild discomfort and fever throughout the body;
(3) Conscious pain is obvious, but there may be unbearable severe pain or pain left after the rash subsides.

#### 2.2.3 Interventions and comparison

The interventions for the test group must be one of acupuncture methods (include acupuncture and moxibustion), or a combination treatment of any 2 methods, or a combination treatment of 1 acupuncture method and western medicine.

The interventions for the control group is western medicine or a treatment above differ with the test group. Additionally, western medicine treatment must be antiviral therapy in guideline,^[3]^ alone or in combination with other treatments such as corticosteroid therapy, neuralgia therapy, and neurotrophic therapy. Drugs for antiviral therapy include acyclovir, valaciclovir, and famciclovir. Studies with 3 or more intervention methods will not be included in order to eliminate the interference of multiple factors.

### 2.3 Outcomes

The primary outcome is effectiveness rate. Clinically, the effectiveness rate of acupuncture and moxibustion (including the objective ratio of skin lesion area reduction and subjective pain scale) is a commonly used and widely recognized outcome indicator for the efficacy of acupuncture and moxibustion in treating herpes zoster. Effectiveness rate was mainly reported by measuring symptom improvement according to Standards for Diagnosis and Curative Effect of Chinese Medical Symptom. The effectiveness rate is calculated as the ratio of the number of patients treated effectively to the total number of patients. Effective treatment includes those who have cured or shown improvement. According to Standards for Diagnosis and Curative Effect of Chinese Medical Symptoms, evaluation criteria are defined as 3 levels,^[20]^

(1) Cured: the rash disappeared, clinical symptoms disappeared, no pain sequela;
(2) Improvement: the rash subsided by more than 30%, and the pain was significantly reduced;
(3) Inefficacious: the rash subsided by less than 30% and pain was not relieved.

The second outcome is analgesic effect evaluation: visual analog scale (VAS) was used for evaluation, 0 means no pain and 10 indicates extreme pain. VAS scores should be recorded before and after treatment. The analgesic efficacy evaluation is calculated by nimodipine method, that is, the efficacy index = (score before treatment − score after treatment)/score before treatment × 100%. Cure: the pain disappears completely; Obvious effect: the pain was significantly reduced compared with that before treatment, with the efficacy index ≥60%; effective: the pain was less than before treatment, 20% < efficacy index < 60%; invalid: the pain is less than before treatment, the curative effect index ≤20%.

### 2.4 Study selection and data extraction

Endnote X9 and Excel 2018 will be used for study selection and data extraction. First, 2 independent investigators will screen titles and abstracts after removing duplicate studies in Endnote. Second, they will read the full-text of relevant studies after titles-abstracts screen, according to inclusion and exclusion criteria. Any discrepancies should be resolved by the third investigators. Finally, included studies will be coded and extracted the relevant information^[19]^: study characteristics (author, publication time); participant characteristics (diagnose criteria, age, disease course, cases); intervention information (intervention detail, treatment duration, follow-up, adverse events), and treatment outcome.

### 2.5 Study quality evaluation

According to Cochrane risk of bias assessment tool,^[19,21]^ the risk of bias will be assessed by 2 investigators independently using Review Man 5.3. The following aspects will be evaluated^[21]^:

(1) Random sequence generation;
(2) Allocation concealment;
(3) Blinding of participants and personnel;
(4) Blinding of outcome assessment;
(5) Incomplete outcome data;
(6) Selective reporting;
(7) other bias.

Any discrepancies would be decided by other investigators within the review team.

### 2.6 Statistical analysis

Firstly, global inconsistency and local inconsistency test and network graphs will be conducted using Stata 13.0 with the network and network graphs packages.

Secondly, network meta-analysis will be conducted in a random-effects model using a Bayesian framework in generate mixed treatment comparisons 0.14.3 with Markov Chain Monte Carlo and further analysis with Stata 13.0.^[19]^ Brooks–Gelman–Rubin diagnosis plot and the potential scale reduced factor are used to evaluate the convergence of the model.^[19,22]^ The odds ratio were calculated for dichotomous outcomes (effectiveness rate) with 95% confidence interval. Numerical variables (pain VAS scores) will be presented as standardized mean difference with a 95% confidence interval.

Finally, we will detect publication bias using the funnel plot and Egger test.

## 3 Discussion

In traditional Chinese medicine, shingles is called “snake string ulcer,” which is caused by accumulation of pathogenic toxin, emotional paralysis, and poor circulation of qi and blood in the viscera. Acupuncture and moxibustion therapies are often considered to have the function of clearing local qi and blood, activating channels and collaterals, and enhancing the ability to resist pathogenic factors, so as to prevent the spread of the virus and alleviate neuralgia. However, the efficacy of different acupuncture and moxibustion methods lacks detailed comparison and ranking. This Bayesian network meta-analysis would represent the most comprehensive synthesis of data for currently acupuncture and moxibustion treatments for herpes zoster. It will incorporate the direct comparison and indirect comparison of various acupuncture and moxibustion therapies. This review will provide a possible ranking for acupuncture and moxibustion treatments for herpes zoster and find a better selection guideline for clinicians and patients. This protocol has been registered with international prospective register of systematic reviews (https://www.crd.york.ac.uk/prospero/).

## Author contributions

L Deng and HC Xu conceived and designed the study. HC Xu, L Deng and YC Shi will search and select the articles. HC Xu, P Liu and YC Shi will extract and analyze the data. HC Xu and L Deng will interpret the data and write the manuscript.

## References

[1] Bricout H Haugh M Olatunde O Herpes zoster-associated mortality in Europe: a systematic review. Bmc Public Health 2015;15:466. 25940080 10.1186/s12889-015-1753-y PMC4435558

[2] Dworkin RH Johnson RW Breuer J Recommendations for the management of herpes zoster. Clin Infect Dis 2007;44:S1–26. 17143845 10.1086/510206

[3] Gross G Schofer H Wassilew S Herpes zoster guideline of the German dermatology society (ddg). J Clin Virol 2003;26:277–89. 12637076 10.1016/s1386-6532(03)00005-2

[4] Robert J Bibhat M David B Guidelines for the management of shingles. Report of a working group of the British society for the study of infection (bssi). J Infect 1995;30:193–200. 7673741

[5] Edmunds WJ Brisson M Rose JD The epidemiology of herpes zoster and potential cost-effectiveness of vaccination in England and Wales. Vaccine 2001;19:3076–90. 11312002 10.1016/s0264-410x(01)00044-5

[6] Yawn BP Gilden D The global epidemiology of herpes zoster. Neurology 2013;81:928–30. 23999562 10.1212/WNL.0b013e3182a3516e PMC3885217

[7] Yawn BP Saddier P Wollan PC A population-based study of the incidence and complication rates of herpes zoster before zoster vaccine introduction. Mayo Clinic Proc 2007;82:1341–9. 10.4065/82.11.1341 17976353

[8] Johnson RW Wasner G Saddier P Postherpetic neuralgia: epidemiology, pathophysiology and management. Expert Rev Neurother 2007;7:1581–95. 17997705 10.1586/14737175.7.11.1581

[9] Mallick-Searle T Snodgrass B Brant JM Postherpetic neuralgia: epidemiology, pathophysiology, and pain management pharmacology. J Multidiscip Healthc 2016;9:447–54. 27703368 10.2147/JMDH.S106340 PMC5036669

[10] Gater A Abetz-Webb L Carroll S Burden of herpes zoster in the UK: findings from the zoster quality of life (ZQOL) study. BMC Infect Dis 2014;14:402. 25038799 10.1186/1471-2334-14-402 PMC4223600

[11] Roxas M Herpes zoster and postherpetic neuralgia: diagnosis and therapeutic considerations. Altern Med Rev 2006;11:102–13. 16813460

[12] Liu ZS Peng WN Liu BY Clinical practice guideline of acupuncture for herpes zoster. Chin J Integr Med 2013;19:58–67. 23275016 10.1007/s11655-013-1191-y

[13] Lee MS Ernst E Acupuncture for pain: an overview of Cochrane reviews. Chin J Integr Med 2011;17:187–9. 21359919 10.1007/s11655-011-0665-7

[14] Ursini T Tontodonati M Manzoli L Acupuncture for the treatment of severe acute pain in herpes zoster: results of a nested, open-label, randomized trial in the vzv pain study. BMC Complement Altern Med 2011;11:46. 21639941 10.1186/1472-6882-11-46 PMC3125389

[15] Coyle ME Liang H Wang K Acupuncture plus moxibustion for herpes zoster: a systematic review and meta-analysis of randomized controlled trials. Dermatol Ther 2017;30:e12468. 10.1111/dth.12468 28338265

[16] Quan XH Li SQ Ma H Different moxibustion methods for herpes zoster: a systematic review and meta-analysis. China J Tradit Chin Med Pharm 2017;32:3768–73.

[17] Yu XM Zhu GM Chen YL Systematic assessment of acupuncture for treatment of herpes zoster in domestic clinical studies. Zhongguo Zhen Jiu = Chin Acupunct Moxib 2007;27:536–40. 17722838

[18] Wang JH Chen HP Chen J Systematic evaluation of a randomized controlled trial of fire acupuncture for the treatment of herpes zoster. J Clin Acupunct Moxib 2009;25:16–8.

[19] Xu HC Shi YC Xiao YK Efficacy comparison of different acupuncture treatments for primary insomnia: a Bayesian analysis %j evidence-based complementary and alternative medicine 2019;2019:13. 10.1155/2019/8961748 PMC6745175 31565065

[20] Cochrane Handbook for Systematic Reviews of Interventions Version 5.1.0 [updated March 2011]. In: Higgins J, Green S, eds. www.cochranehandbook.org: The Cochrane Collaboration; 2011.

[21] State Administration of Traditional Chinese Medicine. Standards for diagnosis and curative effect of Chinese medical symptom. Nanjing: Nanjing University Publishing House, 1994:259.

[22] Brooks SP Gelman A General methods for monitoring convergence of iterative simulations. J Comput Graph Stat 1998;7:434–55.

